# Microbial Features Indicating the Recovery of Soil Ecosystem Strongly Affected by Mining and Ore Processing

**DOI:** 10.3390/ijerph18063240

**Published:** 2021-03-21

**Authors:** Zuzana Feketeová, Andrej Hrabovský, Ivan Šimkovic

**Affiliations:** Department of Soil Science, Faculty of Natural Sciences, Comenius University in Bratislava, Mlynská dolina, Ilkovičova 6, 84215 Bratislava, Slovakia; andrej.hrabovsky@uniba.sk (A.H.); ivan.simkovic@uniba.sk (I.Š.)

**Keywords:** soil microorganisms, microbial indicators, toxic metals, tailings derived soils, recovery

## Abstract

Tailings-derived soils formed from waste materials produced during mineral processing often exhibit extremes of pH, low content of organic matter and limited nutrient availability. The success of site revitalization depends mostly on the ability to maintain natural soil functions. We analyzed technogenic sediments from four selected localities in Slovakia defined as environmental burdens: Slovinky (SLS, SLD), Markušovce (MAS, MAD), Lintich (LIS, LID), Horná Ves (HVS, HVD) in the presented research. None of these sites has long been used for its original purpose. In all localities, the concentrations of several risk elements (As, Ba, Cd, Co, Cr, Cu, Ni, Pb, Zn) still significantly exceed the statutory limit values. Besides the content of risk elements, the amounts of organic carbon, total nitrogen, pH value and moisture level in technogenic substrates were determined. We evaluated selected microbiological parameters, including microbial biomass carbon (MBC), microbial respiration and cellulolytic activity to determine how soil organisms tolerate long-term pollution. In general, the values of microbiological parameters were not as low as one would expect. The results confirmed a negative correlation between MBC content and concentrations of several toxic metals (Co, Cr, Cu, Ni, Zn). The values of assessed microbial indicators were in several cases comparable to those in natural soils. We noticed the lowest metabolic quotient values (qCO_2_) in the heavily polluted locality HVS. The microbial quotient (qMic) was low in every locality except HVS, where the substrate availability index (SAI) was highest. The soil microbial community properties have shown that, despite adverse conditions, these emerging soils allow the growth and development of microorganisms to such an extent that they can adequately use available (although limited) nutrients. The data obtained suggest that these severely impacted soil ecosystems can restore their original environmental functions in time.

## 1. Introduction

Industrial activities modify the landscape and may cause severe environmental impacts. In this context, the areas with a degraded quality of the environment are often referred to as environmental burdens. Mining is among the human activities that significantly impact the ecosystems because it severely modifies the local landscape due to the extraction, processing, storage, transport, or deposit of barren wastes and tailings, bringing about changes in the soil, which becomes challenging to rehabilitate [1]. Therefore, these industrial deposits remain as testimony to the industrial past and can serve as environmental archives of industrial impact. Approximately 50 tailings ponds from the ores and power processing are known in Slovakia (Central Europe). Some of them are reclaimed or suspended, others are in the ore leaching intensification period, and a large part is in regular operation. Most of the geomaterials deposited in tailings ponds are power plant and heating plant waste (furnace slag and fly ash), or ore treatment products (flotation sludge). Such tailings have the character of anthropogenic sediments and pose a potential threat to the environment, as they are a possible source of many toxic elements [2]. As a result of leakage of sludge, large amounts of contaminants enter the soil, the most important of which are toxic metals and other potentially toxic elements that reduce plant production and affect the soil microbial community. Usually, these sites are derelict areas that need to be reclaimed because of the high contaminant levels. Disposal sites (e.g., tailings ponds) are often covered with vegetation due to spontaneous succession or are reclaimed to re-establish an ecological balance in a degraded area. This leads to plant cover formation and stimulating soil-forming processes and initiating biological activity on disposal site surfaces. As a consequence, technogenic soils are being formed, providing habitats for organisms on the superficial parts of tailings ponds [3]. 

Technosols (including those developing on industrial or urban wastes) are closely associated with the heterogeneous sediments of human origin. They provided limited ecosystem services due to potential organic or inorganic contamination and unfavourable physical properties. However, organisms like microarthropods, microorganisms and selected plant species have been reported to inhabit even heavily polluted soils [4,5,6,7]. Thus, soil biota activity in Technosols is contributing to the genesis of these soils and the services they are providing. Favourable climate, young age of the soils and their parent materials’ disequilibrium suggest a rapid evolution of pedogenic processes that in technosols generally appear to progress faster than in natural soils [8]. Little is known about the pedogenic processes that affect technogenic materials, mainly because of their high heterogeneity and a short period of soil genesis. Accumulation of organic matter at the soil surface is often reported as the primary pedogenic process in young technosols [9]. Microorganisms utilize organic matter as a source of energy, which enables their growth and procreation in soil. The litter decomposition process and humus formation are linked with microbial activities that modify the chemical structure of the soil organic matter (SOM) as perceived in nutrient immobilization [10]. Moreover, microbial biomass transformation influences the stability of soil structure and soil fertility [11]. Due to their widespread distribution, rapid growth, metabolic diversity and adaptation to extreme conditions, microorganisms play a central role in soil evolution and formation [12]. Thus, it is clear, that the initial processes of soil formation and input of nutrients rely on the soil microorganisms´ activity and therefore verifying soil attributes in these areas is of great importance for evaluating rehabilitation processes after industrial activities [13].

Soil biological properties are affected by nutrient management and respond quickly to imposed changes. Additionally, a strong relationship was reported among microbial biomass, soil respiration and enzymatic activities [14]. In some highly contaminated soils, organisms are active because metals are not entering their biomass, or the organisms have developed some adaptation towards increased concentration of metals. Therefore, it is essential to identify stable indicators that will report harmful changes in the environment. A variety of microbial parameters have the potential for use as diagnostic indicators of environmental risk. The metabolic characteristics of soil microbial communities are known to be sensitive to management [15] and may also provide information on the status and activity of the microbial community and the resilience of the community to stress [16]. Microbial respiration is usually an essential component of total soil respiration and occurs during organic matter decomposition. Soil respiration is conditioned by humidity, but mostly by ambient temperature. Microbial respiration is the most critical factor reflecting the availability of SOM and intensity of its decomposition [17]. Hence the accumulation of organic carbon in the surface layers of the sludge material and microorganisms’ activity is crucial in soil development in the mining areas [18]. Static soil quality indicators, such as the content of SOM or the C/N ratio, allow us to recognize the long-term changes taking place in the soil. In contrast, dynamic indicators, such as microbial respiration, soil microbial biomass or enzymatic activity, are very sensitive to even the smallest changes and, therefore, knowledge of these characteristics can give us answers even after a short time of stress factor [19]. Thus, the investigation of soil biota and their activity concerning other ecosystem components, especially soils, is essential for a better understanding of the formation and the functioning of these new ecosystems and can consequently be used to design sustainable management solutions for these sites [20].

This study contributes to evaluating soil quality in these areas by integrating soil biological indicators with chemical properties. The hypothesis is that soil microbiological indicators are sensitive in detecting significant positive environmental changes in an area rehabilitated after industrial activities, that can provide biologically relevant insights on new soil-forming processes and present the great potential to predict the restoration of the environmental functions.

## 2. Materials and Methods

### 2.1. Study Area

**Figure 1:**
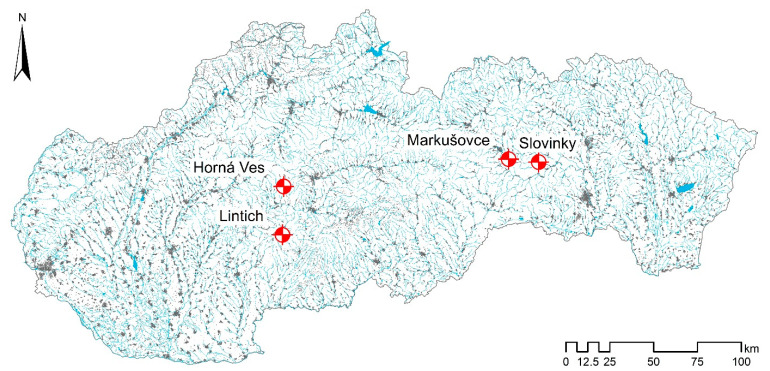
Location of sampling areas in the map of Slovakia: Slovinky (SLS-slag, SLD-dam), Markušovce (MAS-sludge, MAD-dam), Lintich (LIS-sludge, LID-dam), Horná Ves (HVS-sludge, HVD-dam).

This investigation was aimed to study the soil microbiological properties and related chemical characteristics in soils forming at tailings ponds. Four different areas located in Slovakia (Central Europe) (Figure 1) were selected.

**Figure 2:**
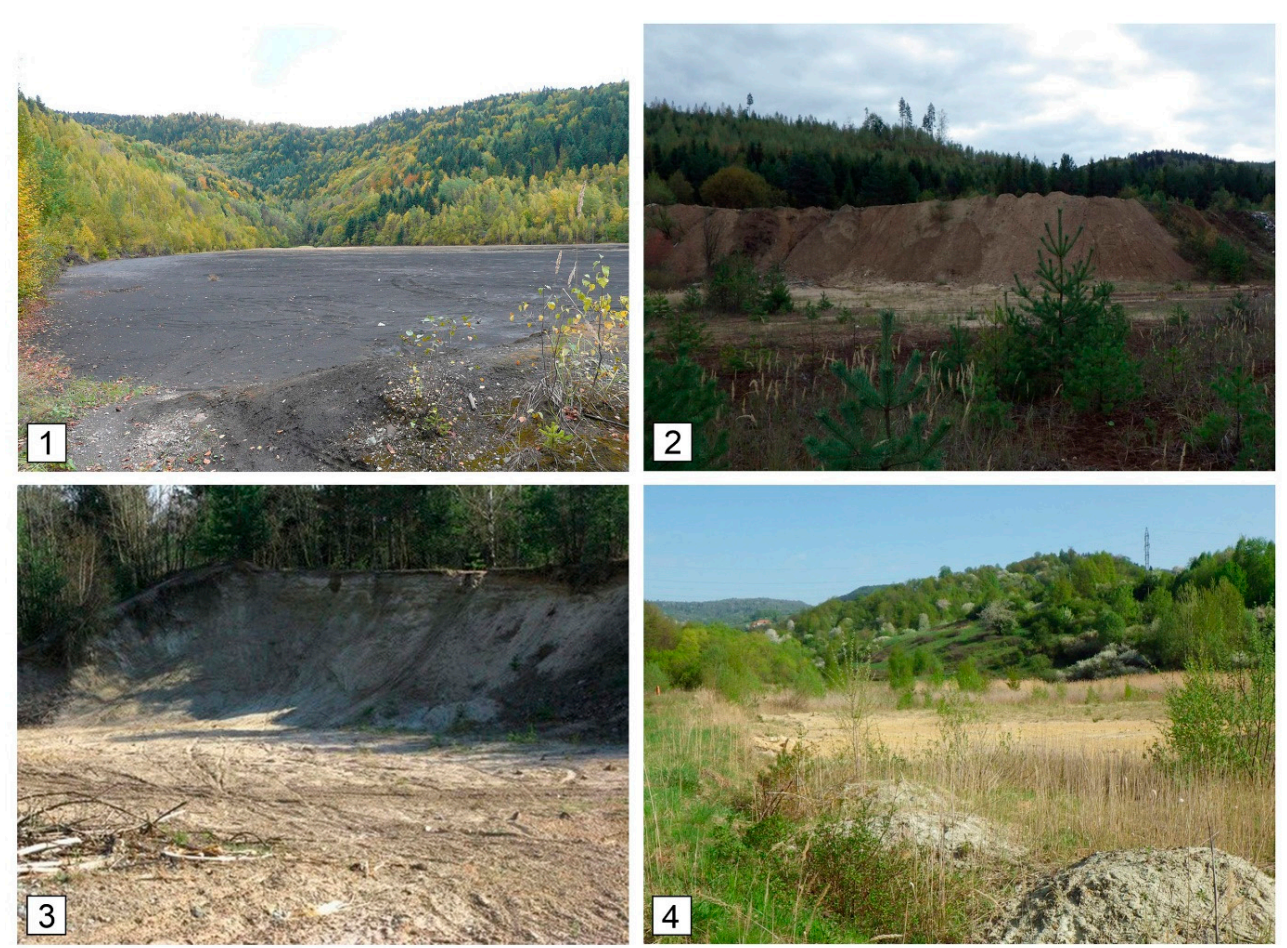
Images show the restored tailings ponds: 1—Slovinky (SLS, SLD), 2—Markušovce (MAS, MAD), 3—Lintich (LIS, LID), 4—Horná Ves (HVS, HVD). SLS—Slovinky slag; SLD—Slovinky dam; MAS—Markušovce sludge; MAD—Markušovce dam; LIS—Lintich sludge; SLD—Linitch dam; HVS—Horná Ves sludge; HVD—Horná Ves dam.

Even though each of the tailings ponds is subject to different reclamation management, studied materials at all the localities, Slovinky (SLS-slag, SLD-dam), Lintich (LIS-sludge, LID-dam), Markušovce (MAS-sludge, MAD-dam) and Horná Ves (HVS-sludge, HVD-dam), represent wastes from mining and ore processing in the form of sludge (Figure 2).

From the assessed sites, only the Markušovce tailing pond is still active. However, we took the samples from the old part of the reservoir, which is not currently used and is overgrown with vegetation. For the comparison, we also present the microbial parameters for each pond’s dam, although it is an area where the technogenic material was already mixed with the original forest soils. The natural forest soils at individual localities were classified according to the World Reference Base for Soil Resources (WRB) Classification System [21] as follows: Slovinky—Folic Lithic Leptosol; Markušovce—Leptic Podzol Skeletic; Lintich—Eutric Cambisol; Horná Ves—Dystric Cambisol.

#### 2.1.1. Slovinky (SLS-Slag; SLD-Dam)

The tailings pond situated above the village Slovinky is the largest in Slovakia with the height of the dam 113 m. Its upper part forms a continuous layer of fine-grained industrial slag (approximately 5–6 m). The deposited mineral waste originates mainly from the flotation of siderite ore and is stored as flotation sands. The substrate is not inhabited by vegetation, probably due to its toxicity. The tailings pond has not been in operation since 1999, and no remediation interventions have been carried out here. The primary contaminant in the substrate is arsenic, and high amounts were found mainly in the upper layer, to a depth of 5 m [6].

#### 2.1.2. Markušovce (MAS-Sludge; MAD-Dam)

The tailings pond has been used as dumping area for flotation sludge originating from the treatment and processing of siderite-barite-sulphide ores. The altitude at the upper dam reaches 478 m above sea level, and at the lower dam 477 m. The tailings pond has a total length of approximately 1.085 km and a width in the range of 160–340 m. The area of the sludge pond is 35 ha [22]. The old reservoir, at which the work is no longer being carried out, is overgrown spontaneously with vegetation, forming a dense cover of grass and moss, and providing a refuge for a wide range of species of small terrestrial rodents (author´s observation).

#### 2.1.3. Lintich (LIS-sludge; LID-dam)

The Lintich tailings pond fills the area of the crashed reservoir. The basic dam is built at a level of about 470 m above sea level. The entire area of the tailings pond occupies 21 ha. It stores about 585,000 tons of fine-grained sand from the flotation treatment of polymetallic ores. The operation of the tailings pond ended in 1974. An experimental afforestation of the tailing pond took place in late 1970s, which led to restoration of the habitats for animal species. [23]. However, alkaline character and possible salinity of the Lintich sludge manifests itself as a negative factor, as it prevents profitable growth of trees and grass, which was reflected in the afforestation attempt.

#### 2.1.4. Horná Ves (HVS-Sludge; HVD-Dam)

The tailings pond Horná Ves is situated in the valley, approximately 230 m from the nearest dwellings. The sewer pipe, about 4.500 km long (pipe diameter 200 mm-concrete and steel pipes) was placed in steep terrain. The technology (consisting of ore gravity, flotation, amalgamation, and leaching concentrates with cyanide) for processing lean gold-silver ore-anti-current leaching with sodium cyanide (NaCN)-was designed and subsequently tested. The flotation waste was diluted with water and discharged to a sludge pond. The intense storm in the summer of 1971 gradually brought two storm waves which caused the rupture of the dam of the tailings pond. After this accident, an approximately 2 m-thick layer of bentonite was put on the pond surface to stabilize the sludge. Nowadays, the area is spontaneously overgrown with vegetation [24].

### 2.2. Soil Sampling and Analyses

The samples were collected from each site every year during the four-year period. We chose the two areas within the tailings pond: the zone of the tailing deposits and dam of impoundment-a heterogeneous mixture of the impoundment sediment, and the forest soil (each of the ponds is surrounded by natural forest). Soil samples were obtained using soil cores (five soil cores per field from which five samples were combined to give one composite soil sample) from the 0–10 cm depth interval. The samples were immediately transferred to a laboratory. The field-moist samples were passed through a 2 mm sieve. After sieving, part of each sample was refrigerated until microbiological analyses were carried out. All the treatments were performed in triplicates. We measured soil moisture content by the gravimetric method. A subsample of fresh, sieved composite material was weighed, oven-dried (at 105 °C) until there was no further mass loss, and then reweighed. The moisture content was expressed as a mass of water per mass of dry soil.

Soil pH was determined with a glass electrode (Sentix 980, WTW, Weilheim, Germany) in suspension with distilled water and 1 M KCl, respectively, using 1:2.5 weight ratio (20 g of sample, 50 mL of H_2_O or KCl). Apart from pH, redox potential (ORP) and electrical conductivity measurements were performed on selected samples that contained elevated content of risk elements. ORP was measured using platinum Sentix ORP-T 900 electrode (WTW, Weilheim, Germany) in water suspension (with 1:2.5 weight ratio). Similarly, as in the case of pH, the suspension was shaken for 5 min. and measured afterwards. Electrical conductivity was measured in the water-saturated paste with a TetraCon 925 conductivity probe. All three parameters were obtained by WTW 3410 mutimeter (WTW, Weilheim, Germany). The total amount of organic carbon and nitrogen was determined by a method based on the Pregl–Dumas principle using the NCHSO “FLASH 2000” Elemental Element Analyser from Thermo Scientific (Waltham, MA, USA). The analysis of potentially toxic elements in studied samples was carried out in the ACME Analytical Laboratories Ltd. (Vancouver, BC, Canada) by Inductively coupled plasma mass spectrometry ICP-MS. Samples were pre-treated by hot acid digestion. Content of 40 elements was determined (Mo, Cu, Pb, Zn, Ag, Ni, Co, Mn, Fe, As, U, Th, Sr, Cd, Sb, Bi, V, Ca, P, La, Cr, Mg, Ba, Ti, Al, Na, K, W, Zr, Ce, Sn, Y, Nb, Ta, Be, Sc, Li, S, Rb, Hf). The results were confronted with limit values for risk elements defined by the law valid in Slovakia (Act No. 220/2004 for Protection and Utilization of Agricultural Soil by Ministry of Agriculture, Environment and Regional Development of the Slovak Republic).

### 2.3. Determination of Soil Microbiological Parameters

Microbial biomass carbon was measured within a 48 h time interval from sampling, using the fumigation-extraction procedure described by Vance [25]. Briefly, 20 g of the field-moist soil was fumigated with ethanol-free chloroform for 24 h. Soluble organic C was extracted from the fumigated and unfumigated soils using 0.25 M K_2_SO_4_. Soil respiration rate was measured as carbon dioxide evolution over 24 h, according to Schinner [26]. Soil samples (20 g dry matter) were weighed into perforated centrifuge tubes and placed into a screw bottle (250 mL) in the presence of 0.025 N NaOH, which trapped CO_2_ evolved during 24 h preincubation period. The actual measurement started by adding precisely 20 mL of 0.025 N NaOH. After 24 h the soil was taken out of the bottle and titrated with 0.025 N HCl. To determine the intensity of cellulose decomposition, we used a modified Christensen method [27], in which the aerobic decomposition of cellulose is monitored under laboratory conditions in Petri dishes. The sample is sieved through a 2 mm sieve and moistened in a Petri dish. Subsequently, three strips of filter paper (cellulose) are applied to the soil surface. The samples are incubated in the dark at room temperature. It is important to check the soil moisture regularly during the incubation. The amount of decomposed cellulose is determined at the end of incubation, substituted into the appropriate formula, and expressed the intensity of cellulolytic activity (Ac) as % of the decomposed cellulose per unit time. We calculated the microbial metabolic quotient as the amount of basal carbon dioxide produced per unit of microbial biomass carbon [28]. Potential to the basal production of carbon dioxide ratio (CO_2_-P/CO_2_-B) was derived to evaluate the substrate availability index (SAI) according to Cheng [29]. The percentage of the microbial biomass, which is indicative of the changes in the availability of nutrients in the soils studied, was determined from the microbial biomass carbon to total organic carbon ratio MBC/Corg. Presented microbial parameters are the mean of triplicate determinations and are expressed with respect to weight of oven-dry soil.

### 2.4. Statistical Analyses

Empirical data were treated statistically using the programme PAST version 4.03 [30]. Pearson correlation coefficients between soil microbial properties and toxic metal concentration values were calculated. Differences at *p* < 0.05 level were considered significant.

## 3. Results

We found increased concentrations of several risk elements in all evaluated substrates. In the sediment of the Slovinky tailings pond (SLS) and the dam (SLD), each of the detected elements exceeded the maximum permitted concentration of such an element stipulated by Act No. 220/2004. At the Markušovce tailings pond (MAS, MAD), it was mainly the arsenic, barium, and nickel found here in above-limit concentrations. Five elements exceed these values in the Linitch tailings pond (LIS, LID)-barium cadmium, copper, lead and zinc, similarly to the Horná Ves tailings pond (HVS, HVD), where we also detected cobalt in above-limit concentrations (Table 1).

All monitored sediments show a weakly alkaline to alkaline pH, except for the HVD, where we recorded a strongly acidic soil reaction, pH = 3.76 ± 0.32 on the dam, which reached an extreme pH value = 1.85 ± 0.18 in the sludge HVS (Table 2). Detected pH values and the results of ORP and electrical conductivity measurements showed a substantial difference in chemical properties between tailings pond at Horná Ves and sludges at three remaining sites. The material from Horná Ves exhibited the lowest pH (≈2), highest ORP value (480 mV), and markedly higher electrical conductivity (11 260 μS/cm) in comparison to other samples. On the other hand, tailings from three remaining areas showed alkaline pH, with ORP values ranging from 185 (Slovinky) up to 280 mV (Lintich). Moreover, significantly lower conductivities of sludge materials were detected at these three sites, respectively: 196 (Slovinky), 290 (Markušovce), and 332 μS/cm (Lintich). The values suggest that the risk of mobilization of detected toxic elements from the sludge materials and adverse effect on biota is in the case of Horná Ves locality significantly higher as it is at the other sites. The total organic carbon values were medium to high in the evaluated samples, except for the LIS, where we recorded a meagre percentage of the parameter. At the same time, however, we recorded the highest mineralizable nitrogen values from all monitored samples in the dam HVD sample. The carbon to nitrogen ratio, which is an essential indicator of the decomposition rate of SOM, was high in most substrates (Table 2).

**Figure 3:**
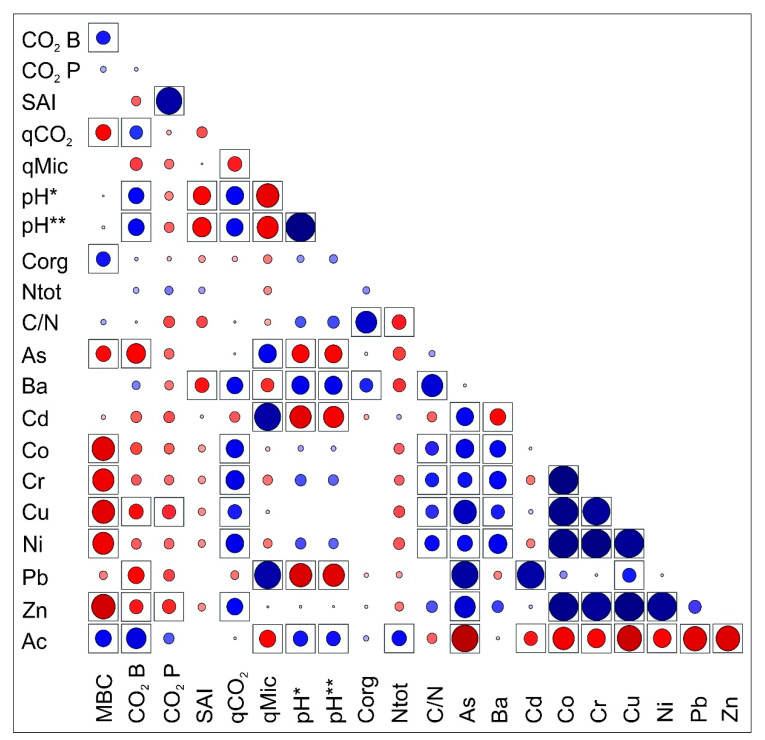
Correlation matrix describing the relations between microbial and chemical parameters of analyzed samples (blue dots represent positive correlation, and red ones represent negative; the bigger and darker is a dot, the strongest relationship between parameters was observed, dots in the box are indicating the significance at *p* < 0.05 level (pH * = pH(H_2_O), pH ** = pH (KCl)).

The only exception was the LID, where a more favorable ratio between carbon and nitrogen was found, which is a surprise as, despite the afforestation performed at this place, the plants are still not doing very well on the site. Correlation analysis showed a strong positive correlation (r = 0.79, *p* < 0.05) between high C/N ratio and high concentration of barium in the substrate (Figure 3), which were found in all the samples except the locality Horná Ves (Table 1).

The microbial biomass carbon values were lowest in the SLS, which, due to its low moisture 12,11 ± 1.99 % and high concentrations of various toxic metals, represents very harsh substrate for microbial community. In other localities (SLD, MAS, MAD, LIS, LID, HVS, HVD), the values of the aforementioned parameters were relatively well proportioned. The MAD sample differed significantly. In this sediment, microbial biomass values were significantly higher than in other samples. We recorded the lowest basal respiration values at the HVS and SLS sites, respectively (including their dams). We also recorded slightly higher values at the LIS. The sample HVD showed the highest values of potential respiration. In other areas, the values were likewise. We determined a slightly lower microbial activity in the HVS and SLS samples (Table 2). The amount of MBC decreased proportionally with the concentration of metals. Statistically, we confirmed a negative correlation for the elements cobalt (r = −0.80, *p* < 0.05), copper (r = −0.79, *p* < 0.05), chromium (r = −0.76, *p* < 0.05), nickel (r = −0.74, *p* < 0.05), and zinc (r = −0.84, *p* < 0.05). Zinc, copper and lead correlated negatively with soil basal respiration. The highest correlation however, was found between soil respiration and arsenic (r = −0.77, *p* < 0.05) (Figure 3).

Considering the contents of risk elements in samples, detected MBC values were not very low. The only exception is perhaps the substrate SLS, in which we recorded a wide range of various toxic metals significantly exceeding the limit values and strongly alkaline soil reaction (pH H_2_O/KCl = 8.72/8.32). Basal respiration of soil microorganisms (CO_2_-B) was even more affected (Table 2)**.** It was most apparent in HVS, where basal and potential respiration reached the minimum values of CO_2_-B = 0.009 ± 0.001 mg CO_2_/g; CO_2_-*p* = 0.048 ± 0.04 mg CO_2_/g. The microbial respiration was in this case influenced not only by high concentrations of copper (3792.4 µg/g) and lead (16,054.7 µg/g), but probably also by the soil reaction of the sediment, which was extremely acidic (pH H_2_O/KCl = 2.06/1.85). We confirmed a positive correlation between pH values and intensity of basal soil respiration (r = 0.52, *p* < 0.05).

**Figure 4:**
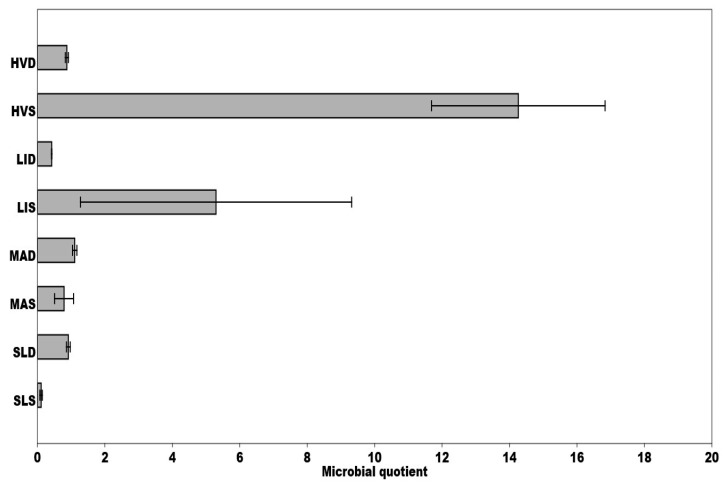
Comparison of mean microbial quotients calculated for each site, during the sampling period, respectively; qMic (MBC/Corg)-the ratio of microbial biomass carbon (mg C/g) to soil organic carbon. Means ± standard deviation (SD) are presented.

The microbial quotient (qMic) often refers to the microbial activity and its potential to mineralize organic matter in the soil. The lower values of this quotient are usually associated with reduced availability of organic material for the microorganisms. The microbial quotient was highest at the HVS and LIS sites, respectively and, conversely, lowest in the case of SLS, SLD, HVD (Figure 4). Moreover, we found a positive correlation between qMic values on one hand and cadmium, or lead concentrations on the other (r = 0.86, *p* < 0.05) (Figure 3). The significantly higher qMic values were detected in the HVS sample.

**Figure 5:**
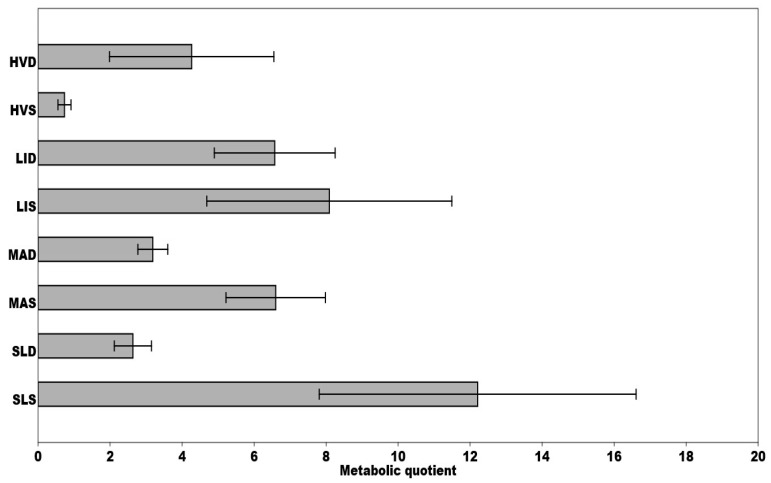
Comparison of mean metabolic quotients, calculated for each site during the sampling period, respectively; qCO_2_ × 100 (CO_2_B/MBC)–microbial respiration (mg CO_2_/g/h) per unit of microbial biomass carbon (mg C/g). Means ± SD are presented.

The metabolic quotient (qCO_2_) is often perceived as useful indicator capturing ecophysiological characteristics and community changes. At the same time, it expresses the efficiency of carbon utilization by the microbial community. Its values increase when the microbial community in the soil is exposed to stress. We recorded the highest values in SLS and LIS samples, respectively. Increased values compared to other monitored substrates were also recorded at MAS and LID sites. Surprisingly, the lowest values were measured at the HVS (the site significantly contaminated by copper, lead, and zinc), as low qCO_2_ values signalize optimal carbon consumption by the soil microorganisms (Figure 5). A positive correlation between qCO_2_ and various toxic elements was also observed (Figure 3).

**Figure 6:**
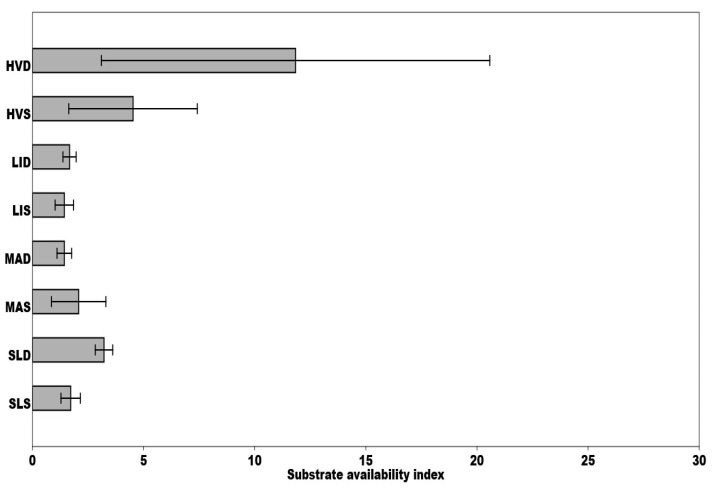
Comparison of mean substrate availability indexes calculated for each sit during the sampling period, respectively; SAI (CO_2_-P/CO_2_-B)–Potential to basal microbial respiration (mg CO_2_/g/h) rate. Means ± SD are presented.

The SAI (substrate availability index) ratio of potential to basal respiration decreases as microbial activity increases, suggesting the amount of readily available organic substances in the substrate. Determined SAI values were low in all the sediments except HVD (Figure 6), which indicates the ability of microorganisms to utilize the readily available organic substances provided by the substrate. Together with the microbial quotient (qMic) values mentioned above, these findings are entirely unexpected. The values of three microbial indicators (SAI, qCO_2_, qMic) were balanced on all monitored areas, except the HVD, where several times higher substrate availability index values were detected (Figure 6). In this pond’s sludge (HVS), we found significantly higher values of the microbial quotient and low metabolic quotient values (Figure 4 and Figure 5).

**Figure 7:**
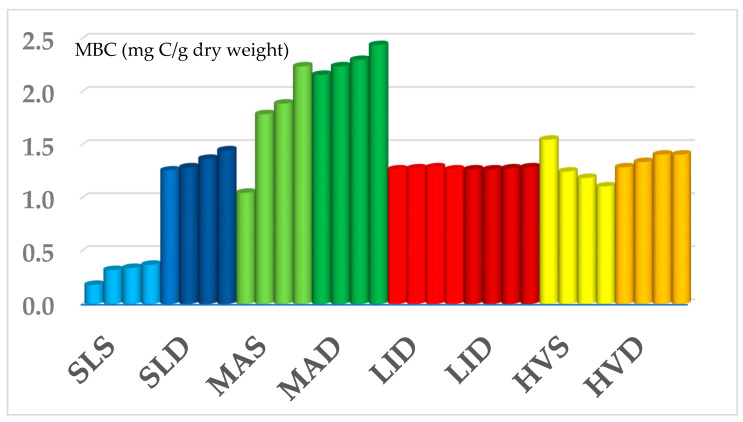
Evolution of microbial biomass carbon (MBC) values (mg C/g) during four sampling seasons on each monitored locality–every color represents a sample: SLS (light blue), SLD (dark blue), MAS (light green), MAD (dark green), LIS (light red), LID (dark red), HVS (yellow), HVD (orange); the average values are shown in the figure for every season, the standard deviation of the means of seasons I–IV were as follows: SLS ± 0.07; 0.08; 0.06; 0.03, SLD ± 0.08; 0.04; 0.13; 0.14, MAS ± 0.04; 0.85; 0.14; 0.07, MAD ± 0.14; 0.13; 0.22; 0.08; LIS ± 0.11; 0.17; 0.11; 0.18, LID ± 0.16; 0.22; 0.12; 0.11, HVS ± 0.33; 0.19; 0.28; 0.18, HVD < 0.01; ±0.04; 0.06; 0.07.

Interestingly, the HVS and SLS sediments did not decompose the cellulose at all, while in other substrates, this proceeded at a slow or moderate pace (MAS Ac = 3, LIS Ac = 2). The substrates´ ability to decompose the cellulose correlated positively with MBC amount and microbial activity. On the other hand, a negative correlation was observed in case of arsenic concentration, which corresponds with the substrates on HVS and SLS sites. In conclusion, it is worth noting that the values of individual monitored parameters have not changed immensely over the years; microbial biomass carbon (Figure 7) or respiration values remain relatively stable.

## 4. Discussion

Several authors have observed increasing respiration in response to the increasing intensity of contamination. They also confirmed that mineralization is inhibited in soils already at values of 1000 mg/kg of zinc, copper or nickel; 100–500 mg/kg of lead and chromium and 10–100 mg/kg of cadmium [31,32]. Chromium is one of the frequently reported elements with an adverse effect on organisms. Many researchers see respiration as the response to negative effects of chromium in the environment [33,34]. Others are of the opinion that it is microbial biomass that can serve as an early indicator. Usually, a rapid decrease in microbial carbon and soil respiration values is recorded shortly after chromium is being added to unpolluted soil, indicating the adaptation of soil microorganisms to contamination [33]. We found high concentrations of chromium in the SLS and SLD sites, respectively (Table 1). Particularly at SLS site the microbial biomass was significantly affected (Table 2).

Considering alkaline pH and a higher content of several risk elements at the SLS site, it cannot be fully distinguished if the toxic effect, manifested through limited activity of microorganisms in the sample, is caused by one element or combination of more elements. When assessing the impact of toxic metals on soil biological properties, we should admit that the lower amount of microbial biomass does not always have to be the result of inorganic pollution. The value of this microbial parameter can naturally decrease during ecological succession. As reported by Tyler et al. [35], soil respiration appears to be unaffected at soil metal levels within the European limits, but at copper or zinc concentrations around 1000 mg/kg, soil microbial activity is reduced. A decrease in the intensity of soil respiration in copper-loaded soils was also noted by El-Ghamry et al. [36] who expressed the assumption that copper significantly affects the growth of plants. In sludge ponds with very high copper concentrations (SLS, SLD, HVS, HVD), we also observed low values of basal and potential respiration, as well as almost total absence of vegetation in the body of the sludge pond (Table 1). Knight et al. [37] observed the effect of cadmium, copper and zinc on the amount of MBC and identified copper as an element that negatively affected this microbial parameter. We found a similar negative relation in the case of copper (r = −0.73, *p* < 0.05). Cadmium also affects microbial metabolic processes and growth. As reported by Fritz et al. [38], even at >25 mg cadmium per kilogram of soil, the intensity of soil respiration decreases. We observed high cadmium concentrations, especially in HVS and SLS, where the intensity of basal and potential respiration was markedly lower as in the other localities. MBC may be affected by other metals like zinc. The results showed that with increase in concentration of zinc the amount of microbial respiration decreased significantly (r = −0.59, *p* < 0.05), similar to the values of MBC (r = −0.84, *p* < 0.05). In contrast, Speir et al. [39] did not observe any significant change of basal respiration and MBC after adding zinc to unpolluted soil. The conclusions of studies aimed on the effects of zinc and cadmium on MBC differ, as a comparison of laboratory and in situ research is not possible, because microorganisms respond differently to the acute and chronic effects of these elements [40].

Most plants are sensitive to toxic metals and are unable to adapt to them. If they appear in the habitat, they show various forms of damage. Plants respond to the metals´ harmful effect in different ways, such as by suppressing photosynthesis, impaired respiration or respiratory dysfunction, so the toxicity of some metals in plants can be manifested by stunted growth and plant chlorosis (yellowing) during full vegetation [41]. The other reason why the sludge is not overgrown with continuous plant cover and the plant (usually different types of grass) in these cases are small and weak, with yellow leaf color, could be the fact that it is difficult for plants to acquire nitrogen at studied sites. Nitrogen-rich substrates with a narrow C/N ratio (ranging from 1–15) are easily degraded compared to substrates with a low portion of N [42]. When an organic substrate has a C/N ratio between 1 and 15, rapid mineralization occurs, and is available for plant intake. The lower the C/N ratio, the more rapidly the nitrogen will be released into the soil. If C/N ratio > 35 results, microbial immobilization occurs [43]. The intensity of cellulose decomposition in contaminated soil is not strictly dependent on overall metal concentration, in comparison to soil properties, which affect the bioavailability of the metal and attenuate its negative impact on soil microorganism. Therefore, the total content of potentially toxic elements in soil is probably not an optimal indicator of microbial degradation of cellulose [44]. We recorded a wide total organic carbon and nitrogen ratio (34.87–151.11) in the tailings where cellulose decomposition did not occur-SLS, SLD, HVS (Table 2). Conversely, in substrates where the C/N ratio was lower (9.31–16.52), the decomposition proceeded rapidly (LIS, LID, HVD). Therefore, in our opinion, the concentration of toxic metals affects the microbial degradation of organic compounds only to a certain extent, and in this case, the limiting factor may be the low humidity, which negatively affects the amount of SOM (considering longer time periods). However, we cannot omit the possibility of the effect of pH on decomposition processes. Slower rates of microbial decomposition in the surface horizon caused by soil acidification in various forest ecosystems of northern and central Europe were reported, for example, by Prietzel et al. [45]. In the long term, the increase of pH towards alkaline values can have an inhibitory effect on decomposition processes as well, leading to lower content of microbial biomass, SOM, and a decrease in cellulolytic activity in soil [46]. Anyhow, we did not statistically confirm a significant relationship between soil pH and cellulose decomposition (Figure 6). Cellulolytic activity (Ac) is one of the essential phases of the decomposition of dead plant matter. It is performed by a diverse group of heterotrophic species, with a significant role of soil microbial community. The intensity of aerobic decomposition of cellulose indicates the ability of microorganisms to decompose organic inputs and also the supply of accessible mineral nutrients in soil. In the samples HVS and SLS, we recorded complete inhibition of cellulolytic activity, partially because the oxides of cobalt and chromium in the soil suppress biodegradation of cellulose [47] and hence reduce the biological activity of the soil [48]. Based on the results of correlation analysis we see that it is mainly arsenic that negatively affects the ability of microorganisms to decompose cellulose.

Although microbial indicators (qMic, qCO_2_ and SAI) have been proposed to indicate the status of a soil ecosystem, it is important to note that none of these characteristics can be used as a universal indicator [49]. Changes in soil biological properties reflect their quality, as soil organisms are much more dynamic and sensitive indicators of pollution than soil’s physical and chemical properties [50]. Metabolic quotient expresses how microbial biomass contributes to the total organic carbon in soil [51]. Microorganisms in less-polluted soils assimilate more carbon and thus produce less carbon dioxide in the process of respiration. In heavily polluted soils, they need more energy to survive, so more carbon is released in the form of CO_2_, as a smaller portion is incorporated into the biomass [52].

By contrast, a very low metabolic quotient proves that, despite the low amounts of organic carbon available to soil microorganisms, the microbial community can use it effectively. Microorganisms can successfully maintain their physiological functions without excessive energy expenditure. Brookes [49] considers the metabolic quotient (qCO_2_) to be a good indicator of the negative effect of inorganic pollution on soil microbiota. The results of his study show that basal respiration values in contaminated soils did not differ significantly, but the metabolic quotient was twice as high. Similar finding was reported by Giller et al. [53], who did not observe significant change in respiration, but microbial carbon values decreased significantly. The data presented in cited works suggest that there is a positive relation between environmental stress, imposed by soil contamination, and metabolic quotient values.

Based on our results, the basal respiration values in assessed areas are comparable to those in the natural environment [54,55]. We also confirmed that MBC values decreased proportionally with increasing concentration of various metals in the samples. In conclusion we found relatively low metabolic quotient values in all monitored sites. A low ratio of basal respiration and microbial carbon indicates a low availability of organic matter for microorganisms and reduced substrate utilization, that leads to long-term microbial biomass suppression in soils. The unexpected low values were determined in one of the most polluted sites, HVS. On the other hand, we can evaluate the microbial indicator as low in all the areas except HVS. Elevated values of this parameter are characteristic, especially for soils in which the pollution persists for a long time and decreases as the magnitude of contamination increases [56,57].

However, all indications are that microorganisms living in such extreme habitats can use very effectively even the small amount of nutrients that the substrate offers. In this case, the substrate probably represents a relatively stable environment comparable to the late phase of succession in the natural ecosystem. An unchanging community of highly adapted species that have adapted to extreme long-term conditions survives without significant problems.

## 5. Conclusions

The impact of industrialization on the natural environment’s quality is evident and, therefore, environmentally friendly strategies for waste management need to be devised. There are several opinions on the effect of toxic metals on microorganisms. Some studies confirm their negative impact, while others argue that there is no correlation between soil microbial properties and increased toxic metal concentrations. These differences are primarily because, in some works, artificially contaminated soil is prepared in the laboratory, while in the others, soil from a loaded habitat is analyzed. However, each of these methods has its pros and cons. We found that the amount of microbial biomass (MBC) decreases proportionally with the concentration of metals. Statistically, we confirmed a negative correlation for the elements-arsenic, cobalt, chromium, copper, zinc, and nickel. Overall, however, we cannot describe the amount of biomass in all studied areas as low. Basal respiration of soil microbial community (CO_2_-B) is meaningfully affected. Decreased cellulolytic activity (Ac), which indicates a microorganisms’ ability to decompose cellulose, was observed in most sediments in which we observed a wider ratio between total organic carbon and nitrogen. As part of data assessment, selected microbial indicators were calculated. We confirmed that sediments of technogenic origin usually show low values of metabolic quotient and substrate availability index (SAI), and at the same time, low values of microbial quotient (qMic). In general, all the specific properties of a microbial community, including the various quotients and indices used to assess the extent of degradation of ecosystems and level of metal pollution, are likely to apply only to a particular environment, in a certain time. In any case, we cannot say with certainty what value already indicates a threat to the soil ecosystem. Increased values of the parameters used may also indicate both pollution and a higher stage of an ecosystem’s succession. We found that, even in substrates significantly polluted with risk elements, the soil microbiota can use available nutrients very efficiently, without unnecessary energy losses or significant difficulties surviving, and even find benefits for its community as well as for the ecosystem. Undoubtedly, polluted ecosystems are capable spontaneously of establishing themselves over time, even at former industrial sites.

## Figures and Tables

**Table 1:** Risk elements concentrations in the samples of studied substrates.

Sample (µg/g)		As	Ba	Cd	Co	Cr	Cu	Ni	Pb	Zn
SLS		428.0	9155.0	2.6	546.0	3678.0	9086.6	565.6	4200.5	25,593.0
SLD		161.0	1103.0	0.9	82.0	1058.0	2236.4	165.0	647.6	6425.0
MAS		141.0	7857.0	<0.5	14.0	89.0	667.8	50.3	11.8	37.0
MAD		47.0	7186.0	<0.5	9.0	72.0	483.8	36.5	2.1	19.0
LIS		10.0	1786.0	13.0	7.0	19.0	324.2	6.3	965.8	2150.0
LID		13.0	1114.0	20.9	7.0	17.0	395.1	7.3	977.1	3202.0
HVS		576.0	251.0	52.0	93.0	16.0	3792.4	28.0	16054.7	9096.0
HVD		117.0	116.0	0.8	2.0	11.0	28.6	4.9	91.9	53.0
	**rv**	**29**	**500**	**0.8**	**20**	**130**	**36**	**35**	**85**	**140**

rv—reference value (µg/g) represents the maximum permissible concentration of a hazardous substance in the soil set by Act No. 220/2004 Protection and Utilization of Agricultural Soil by Ministry of Agriculture, Environment and Regional Development of the Slovak Republic. SLS–Slovinky slag; SLD–Slovinky dam; MAS–Markušovce sludge; MAD–Markušovce dam; LIS–Lintich sludge; SLD–Linitch dam; HVS–Horná Ves sludge; HVD–Horná Ves dam.

**Table 2:** Chemical and microbiological properties of the substrates.

Sample		pH	pH	Corg	Ntot	C/N	MBC	CO_2_B	CO_2_P	w
		(H_2_O)	(KCl)	%	%		mg C/g	mgCO_2_/g	mgCO_2_/g	%
SLS	Mean	8.72	8.32	1.46	0.018	81.11	0.315	0.031	0.064	12.11
	SD	0.09	0.08	0.07	0.007	49.49	0.078	0.009	0.031	1.99
SLD	Mean	8.32	8.19	1.33	0.028	47.50	1.369	0.036	0.112	17.54
	SD	0.08	0.04	0.21	0.007	7.32	0.113	0.007	0.072	0.43
MAS	Mean	8.25	8.13	1.90	0.039	47.50	1.884	0.121	0.082	14.4
	SD	0.22	0.21	0.28	<0.001	6.89	0.840	0.088	0.281	0.43
MAD	Mean	8.45	8.68	3.12	0.034	115.56	2.299	0.073	0.116	13.75
	SD	0.21	0.24	1.47	<0.001	27.23	0.192	0.007	0.029	3.58
LIS	Mean	8.04	7.95	0.42	0.026	16.15	1.270	0.100	0.118	11.00
	SD	0.11	0.11	0.03	0.007	1.09	0.018	0.061	0.047	3.85
LID	Mean	8.09	7.99	1.88	0.202	9.31	1.275	0.089	0.139	6.34
	SD	0.09	0.18	0.98	0.063	4.90	0.016	0.030	0.028	1.61
HVS	Mean	2.06	1.85	1.44	0.039	36.92	1.190	0.009	0.048	20.25
	SD	0.06	0.18	0.15	<0.001	3.81	0.307	0.001	0.039	6.29
HVD	Mean	4.53	3.76	1.29	0.118	11.52	1.370	0.048	0.610	20.94
	SD	0.40	0.33	0.17	0.007	1.51	0.081	0.037	0.172	5.75

pH (H_2_O/KCL)—negative log of the hydrogen ion concentration, Corg—total organic carbon, Ntot—total organic nitrogen, C/N—total organic carbon and nitrogen ratio, MBC—microbial biomass carbon (dry weight), CO_2_B—basal respiration (per 24 h), CO_2_P—potential respiration (per 24 h), w—water content in the sample.

## Data Availability

Data sharing is not applicable to this article. The data are available upon request.
